# The effect of the lockdown executive order during the COVID-19 pandemic in recent trauma admissions in Puerto Rico

**DOI:** 10.1186/s40621-021-00324-y

**Published:** 2021-03-22

**Authors:** Pedro E. Ruiz-Medina, Ediel O. Ramos-Meléndez, Kerwin X. Cruz-De La Rosa, Antonio Arrieta-Alicea, Lourdes Guerrios-Rivera, Mariely Nieves-Plaza, Pablo Rodríguez-Ortiz

**Affiliations:** 1 Trauma Research Program, Department of Surgery, University of Puerto Rico, Medical Sciences Campus, PO Box 365067, San Juan, PR 00967 USA; 2 Puerto Rico Trauma Hospital, PO Box 2129, San Juan, PR 00922 USA; 3 San Juan, USA

**Keywords:** COVID-19, Injury patterns, Lockdown, Pandemic, Trauma

## Abstract

**Background:**

The COVID-19 pandemic led to world-wide restrictions on social activities to curb the spread of this disease. Very little is known about the impact of these restrictions on trauma centers. Our objective was to determine the effect of the pandemic-associated lockdown on trauma admissions, patient’s demographics, mechanisms of injury, injury severity, and outcomes in the Puerto Rico Trauma Hospital.

**Methods:**

An IRB-approved quasi-experimental study was performed to assess the impact of the restrictions by comparing trauma admissions during the lockdown (March 15, 2020 – June 15, 2020) with a control period (same period in 2017–2019). Comparisons were done using the Pearson’s chi-square test, Fisher exact test, or Mann-Whitney U test, as appropriate. A negative binomial model was fitted to estimate the incidence rate ratio for overall admissions among pre-lockdown and during-lockdown periods. Statistical significance was set at *p* < 0.05.

**Results:**

A total of 308 subjects were admitted during the quarter of study for 2017; 323, for 2018; 347, for 2019; and 150, for 2020. The median (interquartile range) age of patients rose significantly from 40 (33) years to 49 (30) years (*p* < 0.001) for the lockdown period compared to the historical period. Almost all mechanisms of injury (i.e., motor vehicle accident, assault, pedestrian, burn, suicide attempt, other) had a slight non-significant reduction in the percentage of patients presenting with an injury. Instead, falls experienced an increase during the lockdown period (18.9% vs. 26.7%; *p* = 0.026). Moreover, the proportion of severe cases decreased, as measured by an injury severity score (ISS) > 15 (37.3% vs. 26.8%; *p* = 0.014); while there were no differences in the median hospital length of stay and the mortality rate between the comparison groups. Finally, the decrease in overall admissions registered during the lockdown accounts for a 59% (IRR 0.41; 95% CI 0.31–0.54) change compared to the pre-lockdown period, when controlling for sex, age, mechanism of injury, and ISS.

**Conclusions:**

Following periods of social isolation and curfews, trauma centers can expect drastic reductions in their overall patient volume with associated changes in trauma patterns. Our findings will help inform new interventions and improve healthcare preparedness for future or similar circumstances.

## Background

On March 11, 2020, the Coronavirus Disease 19 (COVID-19) was officially declared a pandemic by the World Health Organization (WHO) (WHO 2020). At that time, there were no confirmed cases of coronavirus in Puerto Rico and, in a preventive measure, the government declared a “State of Emergency” due to the imminent threat of this disease on March 12, 2020 (Gobierno de Puerto Rico 2020a, 2020b). The following day, the first cases were reported on the island. March 15, 2020, marked the beginning of a new executive order which placed a total lockdown in Puerto Rico (Gobierno de Puerto Rico 2020a, 2020b). All non-essential businesses and social activities were prohibited and a curfew was established to mitigate the effects of this disease. The preventive measures to reduce the spread of this virus included wearing a mask, social distancing, washing hands, and staying at home, among others (Gobierno de Puerto Rico 2020a, 2020b).

The COVID-19 pandemic has irrevocably disrupted our daily lives, which also includes the way we provide essential health services. Due to the anticipated increase in COVID-19 infections, the Centers for Disease Control and Prevention and the American College of Surgeons (ACS) recommended that hospitals and surgeons postpone non-emergent operations to have more resources available to fight this disease (Coronavirus Disease 2019; ACS 2020). However, while non-emergent operations were postponed to accommodate the incoming traumatic injuries and the new logistics challenges presented by the pandemic, no changes in trauma admissions or referral criteria were implemented in our institution, since, as per ACS guidelines, the evaluation of trauma patients should not have been delayed to determine COVID-19 status (ACS 2020). All the above-mentioned actions resulted in a dramatic slowdown of public life in an attempt to “flatten the curve” of the COVID-19 spread (Leichtle et al. 2020). Similar limitations and restrictions were enacted in many countries around the world, some more successful than others (Leichtle et al. 2020).

Trauma, while largely preventable, once it happens cannot be postponed. Despite stringent restrictions on social and public life, patients still experience trauma. Although the COVID-19 pandemic still an on-going issue, the few reports related to its effect on trauma suggest that there has been a considerable reduction in the number of admissions and, in some cases, a change in the mechanisms of injury for which the patients require medical assistance (Leichtle et al. 2020; Kamine et al. 2020; Jacob et al. 2020; Comelli et al. 2020; Nuñez et al. 2020; Christey et al. 2020; Morris et al. 2020; Rajput et al. 2020; Rhodes et al. 2020; Navsaria et al. 2020). The primary objective of this study was to determine the effect of the COVID-19 pandemic and the associated lockdown on trauma admissions, as well as their influence on patient’s demographics, mechanisms of injury, injury severity, and outcomes to the Puerto Rico Trauma Hospital (PRTH). Given the restrictions on public life, which include a stay-at-home order and curfew, we hypothesized there would be a decrease in overall trauma volume and a shift in the mechanisms of injury at the time of presentation. Specifically, we expected a reduction in the most common mechanism at our center, the motor vehicle accidents (MVA), and an increase in non-MVA mechanisms.

## Methods

To accomplish our objective, a quasi-experimental study was conducted at the PRTH (Blume et al. 2020; Su et al. 2020). This 92-bed hospital is the only tertiary institution for polytrauma patients in Puerto Rico and the Caribbean and it is verified as a Level II Trauma Center by the Committee on Trauma of the ACS. Furthermore, our center participates in the US National Trauma Registry System, which allows patient data to be collected in a standardized way and facilitates comparisons with other trauma centers nationwide.

We included in the study all patients who were admitted to the hospital during the lockdown period (from March 15, 2020, through June 15, 2020). As a comparison group, we selected all patients who were admitted during this same quarter in the three preceding years (2019, 2018, 2017). This particular control period was chosen to adjust for seasonal variability (i.e., same quarter) and to provide an unbiased estimator of the expected number of admissions (i.e., multiple control years). Admissions owing to follow-up treatment or complications for a previous injury were excluded.

Patients’ data were retrieved from our trauma registry. Variables of interest included: sex, age, mechanism of injury, type of injury, vital signs (i.e., respiratory rate, systolic blood pressure, and heart rate), Glasgow Coma Scale (GCS), Injury Severity Score (ISS), hospital length of stay (LOS), and in-hospital mortality. All clinical parameters were recorded upon admission to the hospital.

The study population was described employing univariate statistics: mean with standard deviation (SD) or median with interquartile range (IQR) for continuous variables and frequencies and proportions for categorical variables. Comparisons between the lockdown and pre-lockdown periods were conducted using the Pearson’s chi-square test (or the Fisher exact test, as applicable) for categorical data and using the Mann-Whitney U test for continuous variables.

The effect of the COVID-19 related lockdown on overall trauma admissions was modeled using a negative binomial regression with the Puerto Rico annual population estimates specified as the offset term. (Note: 2019 annual population estimates were used for the year 2020, as the 2020 census was not available at the time of analysis.) Negative binomial regression is a generalization of Poisson regression which allows to account for variance inflation. Incidence rate ratios (IRR) with 95% confidence intervals (CI) were calculated unadjusted and adjusted for the following prespecified covariates: sex, age, mechanism of injury, and ISS. Twenty-four (2.1%) patients were missing one or more covariates and, therefore, were excluded from the multivariate analysis.

Finally, time-series plots for weekly admissions were built to compare the number of cases registered during the lockdown period with the average number observed during the previous 3 years. This evaluation was done for all mechanisms in aggregate and separately for the three most prevalent ones. In this analysis, the last 2 days of the period under study were excluded to evaluate the entire weeks.

Our *p*-value criterion for statistical significance was set at 0.05. The statistical software used to perform the analyses was STATA version 14 (STATA Corp, College Station, TX, USA). This study was approved by the Institutional Review Board of the Medical Sciences Campus of the University of Puerto Rico.

## Results

A total of 1128 trauma patients were admitted to the PRTH from March 15 through June 15 from the years 2017–2020. Specifically, 308 subjects were admitted during the quarter of study for 2017; 323, for 2018; 347, for 2019; and 150, for 2020. Sociodemographic and injury-related data is presented in Table 1. The proportion of injured males increased in the lockdown period (77.9% vs. 81.9%), although this difference did not reach statistical significance (*p* > 0.05). Similarly, the median (IQR) age of patients rose significantly during the lockdown compared with the control period from 40 (33) years to 49 (30) years (*p* < 0.001). When evaluating the age categories, an increase in the frequency of admissions of patients aged 35 and above was noted from 57.4 to 74.0%, whereas subjects between ages 15 and 34 frequency dropped from 39.0 to 22.7% (*p* = 0.001).

**Table 1:** Comparison of sociodemographic characteristics, injury profile, and outcomes between pre-lockdown and during-lockdown periods at the Puerto Rico Trauma Hospital

Characteristic	Total (*N* = 1128) n (%)	Pre-Lockdown (*n* = 978) n (%)	Lockdown (*n* = 150) n (%)	*p*-value
***Sociodemographic Data***				
**Sex**	**(*n*** **= 1127)**		**(*n*** **= 149)**	0.273
Male	885 (78.5)	762 (77.9)	122 (81.9)	
Female	242 (21.5)	216 (22.1)	27 (18.1)	
**Age, years**				
Mean ± SD	43.8 ± 20.4	42.8 ± 20.0	49.9 ± 21.7	< 0.001
Median (IQR)	41 (32)	40 (33)	49 (30)	
Categories				0.001
< 15	40 (3.5)	35 (3.6)	5 (3.3)	
15–34	416 (36.9)	382 (39.0)	34 (22.7)	
35–64	461 (40.9)	387 (39.6)	74 (49.3)	
> 64	211 (18.7)	174 (17.8)	37 (24.7)	
***Injury-related Data***				
**Mechanism of Injury**	**(*n*** **= 1122)**	**(*n*** **= 976)**	**(*n*** **= 146)**	
Motor Vehicle Accident	410 (36.6)	360 (36.9)	50 (34.2)	0.537
Assault	245 (21.8)	216 (22.1)	29 (19.9)	0.536
Fall	223 (19.9)	184 (18.9)	39 (26.7)	0.026
Pedestrian	153 (13.6)	134 (13.7)	19 (13.0)	0.814
Burn	17 (1.5)	16 (1.6)	1 (0.7)	0.714^†^
Suicide Attempt	21 (1.9)	19 (2.0)	2 (1.4)	0.999^†^
Other	53 (4.7)	47 (4.8)	6 (4.1)	0.708
**Type of Injury**	**(*n*** **= 1122)**	**(*n*** **= 976)**	**(*n*** **= 146)**	0.451
Blunt	863 (76.9)	750 (76.8)	113 (77.4)	
Penetrating	236 (21.0)	204 (20.9)	32 (21.9)	
Other	23 (2.1)	22 (2.3)	1 (0.7)	
**Respiratory Rate**	**(*n*** **= 1098)**	**(*n*** **= 949)**	**(*n*** **= 149)**	0.807
< 12 bpm	24 (2.2)	20 (2.1)	4 (2.7)	
12–20 bpm	722 (65.8)	622 (65.5)	100 (67.1)	
> 20 bpm	352 (32.0)	307 (32.4)	45 (30.2)	
**Systolic Blood Pressure**	**(*n*** **= 1126)**	**(*n*** **= 976)**		0.698
< 90 mmHg	52 (4.6)	46 (4.7)	6 (4.0)	
≥ 90 mmHg	1074 (95.4)	930 (95.3)	144 (96.0)	
**Heart Rate**	**(*n*** **= 1123)**	**(*n*** **= 973)**		0.625
< 60 bpm	57 (5.1)	47 (4.8)	10 (6.7)	
60 bpm – 100 bpm	686 (61.1)	595 (61.2)	91 (60.7)	
> 100 bpm	380 (33.8)	331 (34.0)	49 (32.6)	
**Glasgow Coma Scale**	**(*n*** **= 1118)**	**(*n*** **= 969)**	**(*n*** **= 149)**	0.233
13–15	923 (82.6)	803 (82.9)	120 (80.5)	
9–12	52 (4.6)	41 (4.2)	11 (7.4)	
≤ 8	143 (12.8)	125 (12.9)	18 (12.1)	
**Injury Severity Score**	**(*n*** **= 1104)**	**(*n*** **= 962)**	**(*n*** **= 142)**	
Mean ± SD	13.7 ± 9.2	13.9 ± 9.2	11.9 ± 9.2	
Median (IQR)	13 (9)	13 (9)	10 (11)	0.002
> 15	397 (36.0)	359 (37.3)	38 (26.8)	0.014
**Hospital LOS, days**	**(*n*** **= 1099)**	**(*n*** **= 957)**	**(*n*** **= 142)**	
Mean ± SD	16.0 ± 22.1	16.0 ± 22.1	16.4 ± 21.9	
Median (IQR)	9 (13)	9 (13)	7 (14)	0.191
**In-Hospital Mortality**	**(*n*** **= 1099)**	**(*n*** **= 957)**	**(*n*** **= 142)**	0.380
Dead	109 (9.9)	92 (9.6)	17 (12.0)	
Alive	990 (90.1)	865 (90.4)	125 (88.0)	

*SD* standard deviation, *IQR* interquartile range, *LOS* length of stay

^†^Fisher’s exact test

MVA were the overall predominant mechanism of injury across all time periods studied. Almost all mechanisms of injury (i.e., MVA, assault, pedestrian, burn, suicide attempt, other) had a slight reduction in the percentage of patients presenting with an injury; however, no statistically significant differences were observed (*p* > 0.05). Falls, on the other hand, experienced an increase (18.9% vs. 26.7%) during the lockdown period compared to the years without the lockdown (*p* = 0.026). Nevertheless, the distributions of the type of injury were independent of the lockdown executive order (*p* > 0.05).

In terms of measurements of trauma severity, the proportion of patients that presented with a high ISS (> 15) was lower during the lockdown period than during the control one (37.3% vs. 26.8%; *p* = 0.014). The GCS, on the other hand, was not significantly related to the lockdown executive order (*p* > 0.05). For the hospital-related outcomes, there was a decrease in the median (IQR) LOS from 9 (13) days in the historical period to 7 (14) days during the lockdown period, although not statistically significant (*p* = 0.191). Similarly, the in-hospital mortality had a slight non-significant increase during the COVID-19 related lockdown (9.6% vs. 12.0%; *p* = 0.380).

The aforementioned measurements of injury severity and outcomes are presented by individual mechanisms of injury in Table 2. In this mechanism-stratified analysis (only the most prevalent ones were included), there were no differences in the median ISS and median LOS between the comparison groups (*p* > 0.05). For in-hospital mortality, pedestrians showed a significant reduction during the lockdown period (21.4% vs. 0%; *p* = 0.043), while the rest of the mechanisms (MVA, assault, and fall) experienced non-significant increase in the proportion of deceased patients.

**Table 2:** Comparison of injury severity and outcomes between pre-lockdown and during-lockdown periods at the Puerto Rico Trauma Hospital, by selected mechanisms of injury

Characteristic	Total	Pre-lockdown	Lockdown	*p*-value
***Motor Vehicle Accident***				
**Injury Severity Score**	**(*n*** **= 400)**	**(*n*** **= 352)**	**(*n*** **= 48)**	
Mean ± SD	14.3 ± 9.1	14.5 ± 9.2	12.3 ± 7.7	0.118
Median (IQR)	13 (9)	13 (9)	10.5 (10.5)	
**Hospital LOS, days**	**(*n*** **= 398)**	**(*n*** **= 350)**	**(*n*** **= 48)**	
Mean ± SD	15.9 ± 20.1	15.6 ± 19.8	18.7 ± 22.5	0.703
Median (IQR)	9 (13)	9 (11)	10.5 (17)	
**In-Hospital Mortality, n (%)**	**(*n*** **= 398)**	**(*n*** **= 350)**	**(*n*** **= 48)**	
Dead	32 (8.0)	25 (7.1)	7 (14.6)	0.088^†^
Alive	366 (92.0)	325 (92.9)	41 (85.4)	
***Assault***				
**Injury Severity Score**	**(*n*** **= 243)**	**(*n*** **= 214)**	**(*n*** **= 29)**	
Mean ± SD	13.2 ± 8.9	13.4 ± 8.7	11.3 ± 9.7	0.064
Median (IQR)	10 (8)	10 (9)	10 (9)	
**Hospital LOS, days**	**(*n*** **= 242)**	**(*n*** **= 213)**	**(*n*** **= 29)**	
Mean ± SD	13.4 ± 17.4	13.3 ± 16.6	13.6 ± 22.6	0.248
Median (IQR)	8 (12)	8 (11)	7 (10)	
**In-Hospital Mortality, n (%)**	**(*n*** **= 242)**	**(*n*** **= 213)**	**(*n*** **= 29)**	
Dead	17 (7.0)	13 (6.1)	4 (13.8)	0.130^†^
Alive	225 (93.0)	200 (93.9)	25 (86.2)	
***Fall***				
**Injury Severity Score**	**(*n*** **= 222)**	**(*n*** **= 183)**	**(*n*** **= 39)**	
Mean ± SD	12.3 ± 7.0	12.5 ± 7.1	11.1 ± 6.0	0.346
Median (IQR)	9 (9)	9 (8)	9 (9)	
**Hospital LOS, days**	**(*n*** **= 221)**	**(*n*** **= 182)**	**(*n*** **= 39)**	
Mean ± SD	13.2 ± 16.6	13.5 ± 17.0	11.8 ± 14.3	0.147
Median (IQR)	8 (10)	8 (10)	5 (9)	
**In-Hospital Mortality, n (%)**	**(*n*** **= 221)**	**(*n*** **= 182)**	**(*n*** **= 39)**	
Dead	21 (9.5)	16 (8.8)	5 (12.8)	0.545^†^
Alive	200 (90.5)	166 (91.2)	34 (87.2)	
***Pedestrian***				
**Injury Severity Score**	**(*n*** **= 148)**	**(*n*** **= 131)**	**(*n*** **= 17)**	
Mean ± SD	15.6 ± 8.7	15.9 ± 9.1	12.9 ± 5.3	0.682
Median (IQR)	14 (11.5)	16 (13)	9 (8)	
**Hospital LOS, days**	**(*n*** **= 148)**	**(*n*** **= 131)**	**(*n*** **= 17)**	
Mean ± SD	23.5 ± 31.2	23.0 ± 31.4	27.4 ± 29.7	0.094
Median (IQR)	12 (23)	12 (22)	16 (37)	
**In-Hospital Mortality, n (%)**	**(*n*** **= 148)**	**(*n*** **= 131)**	**(*n*** **= 17)**	
Dead	28 (18.9)	28 (21.4)	0 (0)	0.043^†^
Alive	120 (81.1)	103 (78.6)	17 (100)	

*SD* standard deviation, *IQR* interquartile range, *LOS* length of stay

^†^Fisher’s exact test

The unadjusted and adjusted IRRs for the overall admissions among our study periods were also calculated (Table 3). There was a 53% reduction in the overall admissions during the lockdown period compared to the historical average for the years 2017–2019 (IRR 0.47; 95% CI 0.38–0.57). Once controlled for sex, age, mechanisms of injury, and ISS, the percent reduction increased to 59% (IRR 0.41; 95% CI 0.31–0.54).

**Table 3:** Unadjusted and adjusted incidence rate ratios for overall admissions among pre-lockdown and during-lockdown periods at the Puerto Rico Trauma Hospital, with corresponding 95% confidence intervals

Overall trauma admissions	IRR (95% CI)	Adjusted IRR^a^ (95% CI)
Lockdown	0.47 (0.38–0.57)	0.41 (0.31–0.54)
Pre-Lockdown (2017–2019)	1	1
Lockdown	0.43 (0.36–0.52)	0.37 (0.27–0.50)
Pre-Lockdown (2019)	1	1
Lockdown	0.46 (0.38–0.56)	0.41 (0.30–0.55)
Pre-Lockdown (2018)	1	1
Lockdown	0.51 (0.42–0.62)	0.43 (0.32–0.58)
Pre-Lockdown (2017)	1	1

Four regression models were built using different reference groups in each one: total admissions during 2017–2019 (mean), 2019, 2018, and 2017

*IRR* incidence rate ratio, *CI* confidence interval

^a^adjusted for sex, age, mechanisms of injury, and injury severity score

### Weekly admissions

**Fig. 1:**
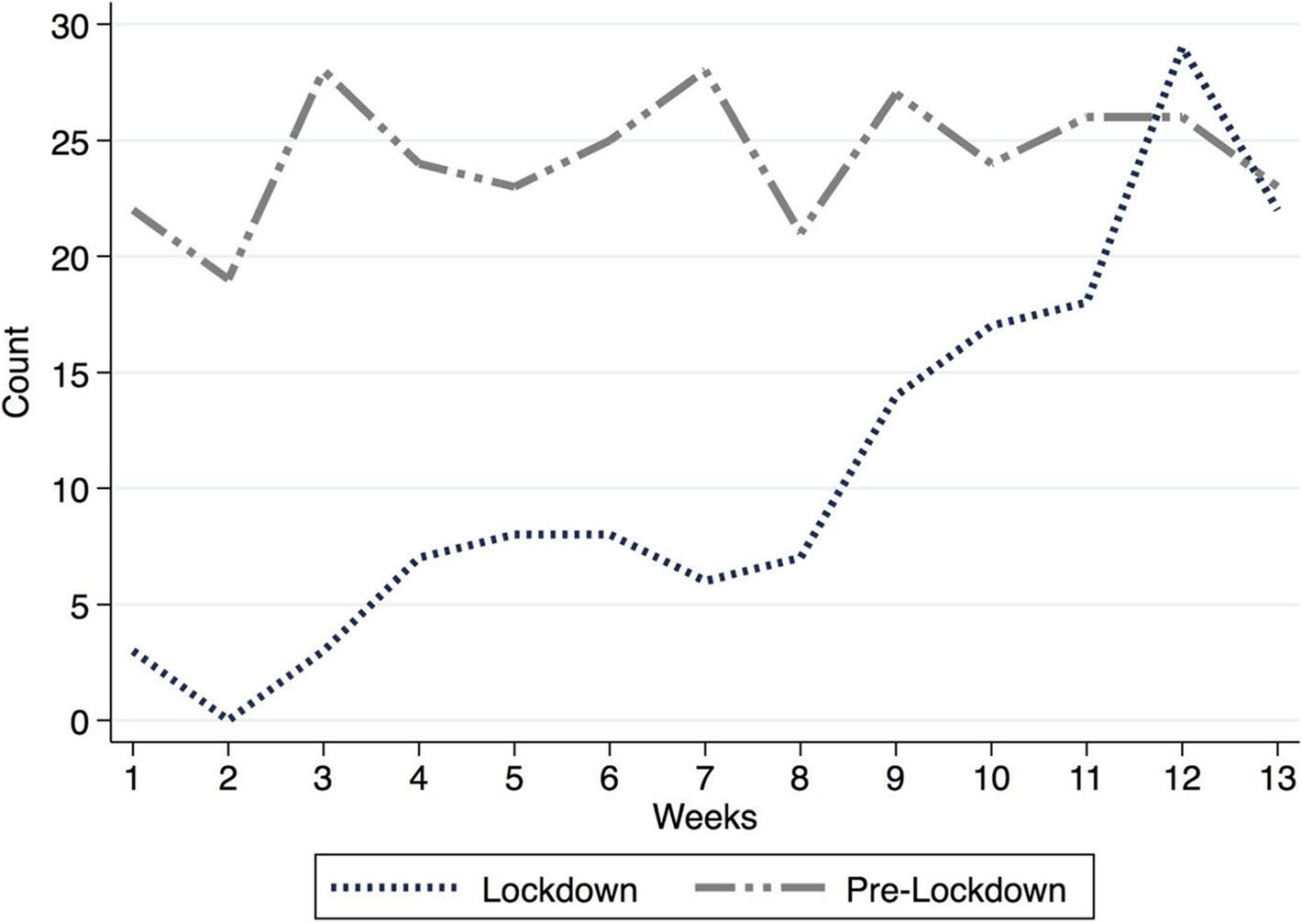
Weekly Admissions for Pre-Lockdown and During-Lockdown Periods. Data for the Pre-Lockdown period is the average of the years 2017–2019

Looking at the admissions breakdown per week, the most substantial hit occurred during the first 8 weeks of lockdown, when admissions did not exceed 10 cases weekly. Indeed, the second week of lockdown represented the lowest possible value with no admissions; coincidentally, the same week for the previous years was also the lowest (on average) in the study period. However, as the lockdown went on, the number of admissions gradually started increasing until eventually reaching the expected value near the end of the quarantine, specifically in week 12 (Fig. 1).

**Fig. 2:**
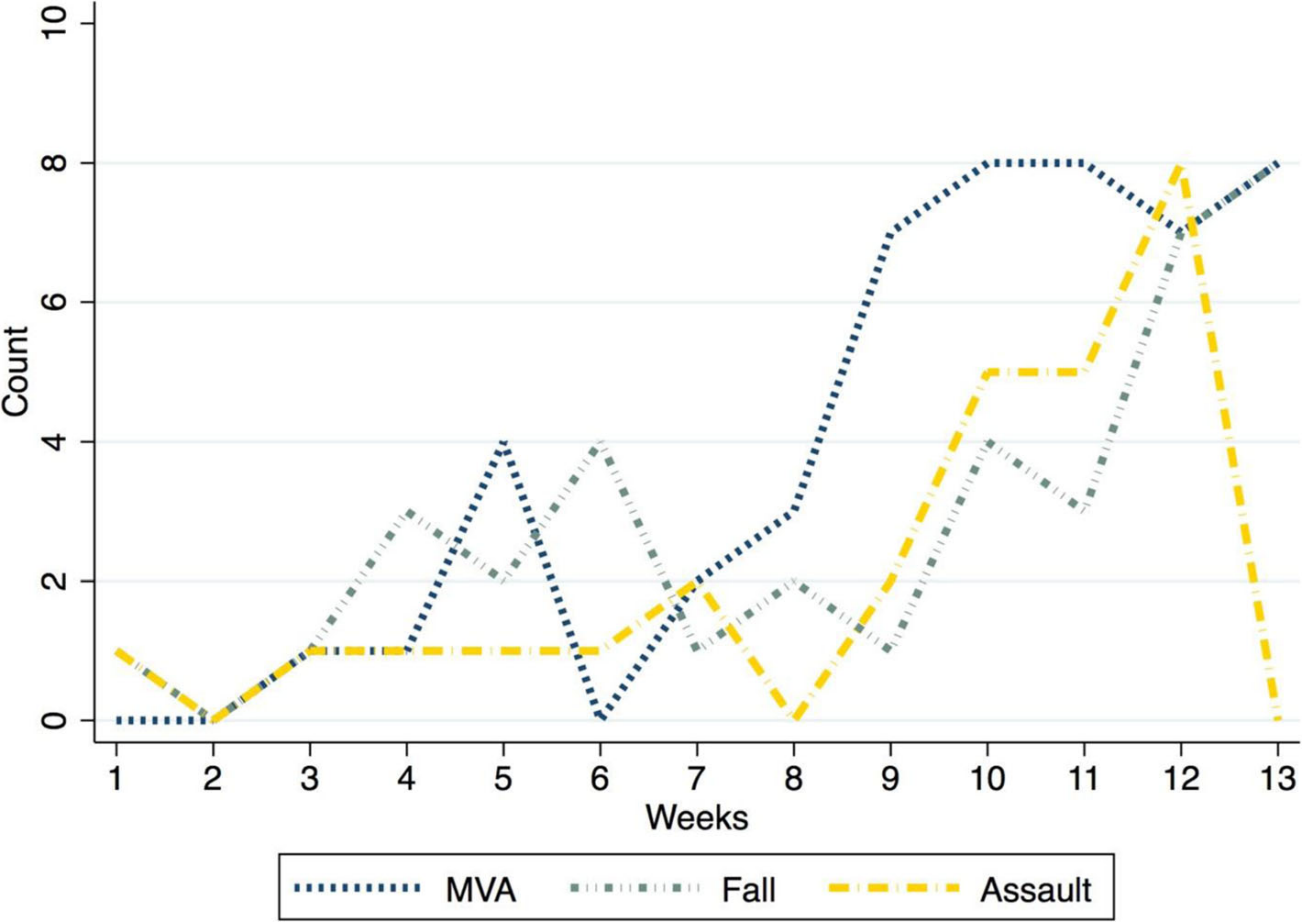
Weekly Admissions for During-Lockdown Period by Selected Mechanisms of Injury

**Fig. 3:**
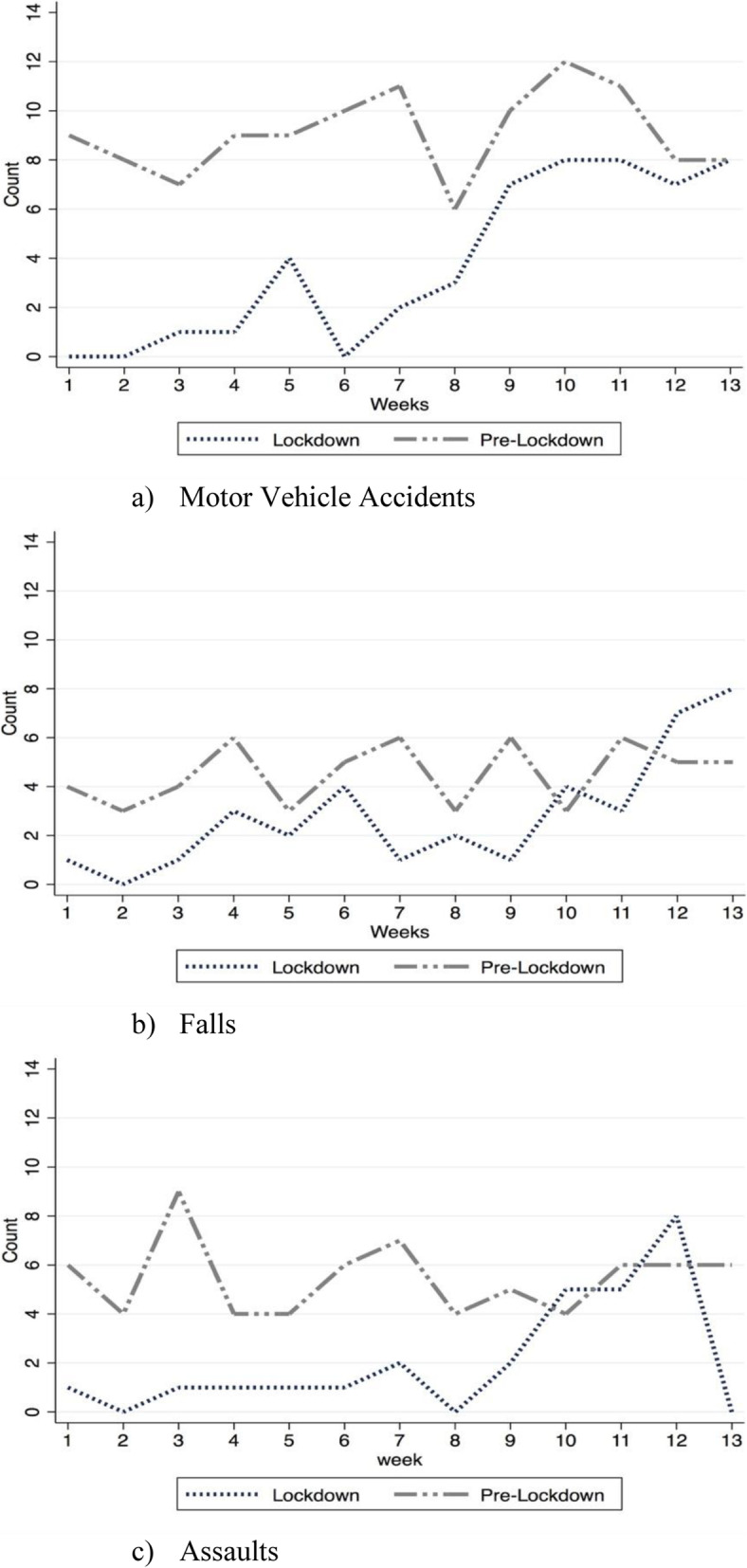
(three panels). Weekly Admissions for Pre-Lockdown and During-Lockdown Periods by Selected Mechanisms of Injury. Data for the Pre-Lockdown period is the average of the years 2017–2019. **a** Motor Vehicle Accidents, **b** Falls, **c** Assaults

Figure 2 shows the weekly admissions during the lockdown period of the three most predominant mechanisms of injury. Looking at the absolute frequency of admissions by week, there were no admissions for MVA during the first 2 weeks after the enactment of the lockdown executive order. Assaults and falls were the prevalent mechanisms of injury during the first month. The comparison of the lockdown period to the pre-lockdown period by the mechanisms of injury is depicted in Fig. 3. For all mechanisms, there is an evident decrease in the number of admissions from the beginning of the executive order. As time goes by, the gap between the pre-lockdown and the lockdown period inches closer until reaching the expected values.

## Discussion

The world-wide imposition, although to varying degrees, of curfew and quarantine as strict measurements to curb the COVID-19 spread, represents the most drastic restrictions to social life in recent times. Data from our study shows that the aforementioned severe restrictions led to the expected decrease in admissions to trauma. The few studies available on the topic, point towards the same downtrend pattern as a result of the paradigm shift caused by the recent changes to our daily lives due to the coronavirus (Leichtle et al. 2020; Kamine et al. 2020; Jacob et al. 2020; Comelli et al. 2020; Nuñez et al. 2020; Christey et al. 2020; Morris et al. 2020; Rajput et al. 2020; Rhodes et al. 2020; Navsaria et al. 2020).

A staggering 59% reduction in the total admissions for the lockdown period compared to the historical period was observed. Similar dramatic decreases in trauma cases, from 37.6 to 57.4%, have been reported worldwide (Leichtle et al. 2020; Kamine et al. 2020; Jacob et al. 2020; Comelli et al. 2020; Nuñez et al. 2020; Christey et al. 2020; Morris et al. 2020; Rajput et al. 2020; Rhodes et al. 2020; Navsaria et al. 2020). Aside from the lockdown and curfew orders, fear of contracting COVID-19 while seeking medical attention, for both traumatic and even non-traumatic injuries, has also been cited as a possible reason for the universal decrease in admissions (Navsaria et al. 2020; Leichtle et al. 2020; Rajput et al. 2020). As anticipated, the first few weeks of the lockdown represented the lowest points of trauma cases per week due to the recently enacted executive orders. It was during that period that the executive order was at its most stringent. However, as time passed by, the number of admissions began increasing until reaching the usual values. As explained by Leichtle et al. (2020), this phenomenon can be explained by “lockdown fatigue” coupled with the gradual easing and liberal interpretations of the subsequent executive orders.

As for the sociodemographic data, males remained the majority of injured patients as was the case in other studies, at 81.9% (Leichtle et al. 2020; Nuñez et al. 2020; Rhodes et al. 2020). The median age of injured patients increased from 40 to 49. Compared to our findings, studies in the past have shown that during periods of natural disasters and forced isolation (e.g., hurricanes, earthquakes, lockdowns), the population who presents to the hospital seeking care tends to be older (Comelli et al. 2020; Ramos-Meléndez et al. 2020).

The injury-related profile of the patients admitted during the lockdown showed a lower ISS (≤ 15) compared to those in the previous years. Other studies have shown either a similar trend or no change at the time of presentation (DiFazio et al. 2020; Jacob et al. 2020). As stated by Morris et al. (2020), if patients were postponing medical care due to the aforementioned fear, the proportion of mild-moderate injury severity would have decreased in comparison with those with more severe injuries. The fact that in our population the ISS was lower while the lockdown executive order was in place, suggests that the discrepancy in trauma admissions should not be attributed to a reduction in health-seeking behavior (Morris et al. 2020). Nonetheless, the reluctance to seek care, which also drives admissions down, has also been observed for other non-surgical conditions, affecting the incidence and mortality of other diseases (Jacob et al. 2020). Worth noting that, while not statistically significant, patients spent fewer days hospitalized and had a slightly higher in-hospital mortality. As suggested by Leichtle et al. (2020), this could potentially be explained by an institutional effort to facilitate rapid discharge to accommodate the rise in covid patients needing to be hospitalized.

In terms of trauma mechanisms, we hypothesized that the lockdown would have a direct impact on the particular mechanisms for which a patient presents to the hospital. Even though people were mandated to stay at home and traffic volume declined, MVA remained the prevalent mechanism of injury during the lockdown, consistent with our data before the pandemic. Similar studies showcase a marked reduction in the percentage of MVA, but in our case, the reduction was not statistically significant (Kamine et al. 2020; Jacob et al. 2020; Comelli et al. 2020; Christey et al. 2020; Morris et al. 2020; Rajput et al. 2020).

Falls, on the other hand, experienced a statistically significant increase in the number of presenting patients compared to the historical period. Along with assaults, falls were the predominant mechanism during the first month of the lockdown. According to the literature, it is among the top mechanisms that increase in frequency in the aftermath of a natural disaster (Ramos-Meléndez et al. 2020). While this is not a natural disaster per se, it led to house confinement usually seen during and after natural disasters. The increase in falls can be explained by the tendency of people to perform activities at home during the lockdown (Rajput et al. 2020).

The majority of the non-MVA mechanisms of injury (assault, pedestrian, burn, suicide attempt, other) suffered a slight reduction (up to 2.2%), although not statistically significant. Most of the studies show either a reduction or no change in terms of these mechanisms, similar to our experience (Leichtle et al. 2020; Kamine et al. 2020; Jacob et al. 2020; Christey et al. 2020; Morris et al. 2020). It is important to note that the difference (either increase or decrease) in the mechanisms of injury at the time of presentation, is most likely related to the geographic region of origin, since there is great variance in the type and duration of the societal restrictions around the world.

This study has some limitations. First, it is a single center study, meaning that our experience may not be extrapolated to other centers. Second, the retrospective nature of the study implies that the data is contingent on the quality of the trauma registry. Particularly, not all data were available for all participants, which might lead to information and selection biases. Despite these limitations, our study has important strengths. The pre-lockdown period included more years than the available studies, which allowed us to provide an unbiased estimator of the expected number of admissions if the lockdown had not been enacted. At the present, this is the first study, to our knowledge, to analyze the effects of social restrictions on trauma admissions in Puerto Rico and the Caribbean and one of the few in the United States as well. The information gathered in this study will allow us, and other trauma centers, to prepare response protocols towards the COVID-19 emergency, which will require coordinated and effective measures to mitigate the ongoing pandemic, as well as similar future health crises. While hospital resource utilization amid a crisis might be institution-specific, experiences such as this one can help guide reallocation efforts (both personnel and equipment) to better serve the patients currently in need. At the same time, the analysis can lead to the development of universal and tailored cost-effective prevention strategies aimed towards the most common mechanisms of injury that prevail during times of social lockdown and the population most affected by them. Specific emphasis should be given to the elderly and male patients when educating the population, while providing effective clinical and community-driven educational strategies to avoid injuries related to falls and motor vehicle accidents.

## Conclusion

As we face one of the worst crises in history, very little is known about its impact on an essential service such as a trauma hospital. The results of our study provide further evidence of the expected marked decrease in the volume of cases that presents in the face of an imposed curfew and lockdown, in our case as high as 59%, which mirrors other international trauma centers. Furthermore, the decrease in admissions is accompanied by changes in patients’ demographics, injury severity (less severe patient presentation) and presenting trauma mechanisms, with falls showing an expected and significant increase during these times of social isolation and state-mandated lockdown. The importance of describing the changes during these trying times lies in the fact that new challenges arise every day, and we must be equipped to quickly adapt, react and, if possible, prevent similar events in the future. Further studies are required to examine the full extent of social restrictions on trauma volume and patterns, as well as long-term outcomes in these patients.

## Data Availability

The dataset that supports the findings of this study is available from the Puerto Rico Trauma Hospital Data Registry but restrictions apply to its availability. Data is however available from the authors upon reasonable request and with permission of the Puerto Rico Trauma Hospital.

## Abbreviations

COVID-19: Coronavirus Disease 19
WHO: World Health Organization
ACS: American College of Surgeons
PRTH: Puerto Rico Trauma Hospital
MVA: Motor vehicle accidents
GCS: Glasgow Coma Scale
SD: Standard deviation
IQR: Interquartile range
ISS: Injury Severity Score
LOS: Length of stay
IRR: Incidence rate ratio
CI: Confidence interval
